# Ningmitai capsule promotes calculi expulsion after RIRS for 10–20-mm upper urinary stones: a multicenter, prospective, randomized controlled trial

**DOI:** 10.1007/s00240-021-01296-w

**Published:** 2022-01-25

**Authors:** Ruofan Wang, Qingdong Qiao, Dengke Yang, Jianguo Zhang, Chaoyang Zhu, Jiantao Sun, Zhongling Dou, Xiaofu Wang, Huiming Zhang, Wenhao Wang, Fei Xiao, Hepeng Cheng, Wenwei Lv, Bo Zhou, Xiaofan Zhang, Wuxue Li, Xinghua Zhao, Bin Hao, Changbao Xu

**Affiliations:** 1 Department of Urology, The Second Affiliated Hospital of Zhengzhou University, No. 2 Jingba Road, Zhengzhou, 450014 Henan China; 2 Department of Urology, Xinxiang Central Hospital, Xinxiang Medical University, Xinxiang, Henan China; 3 Department of Urology, The 990th Hospital of the Joint Logistics Support Force of the Chinese People’s Liberation Army, Zhumadian, Henan China; 4 Department of Urology, The First Affiliated Hospital, and College of Clinical Medicine of Henan University of Science and Technology, Luoyang, Henan China; 5 Department of Urology, Huaihe Hospital of Henan University, Kaifeng, Henan China; 6 Department of Urology, The Luoyang Central Hospital Affiliated to Zhengzhou University, Xigong District, Luoyang, Henan China; 7 Institute of Central Laboratory, The Second Affiliated Hospital of Zhengzhou University, Zhengzhou, Henan China

**Keywords:** Upper urinary calculi, Retrograde intrarenal surgery, Ningmitai capsule, Chinese prescription expulsion

## Abstract

To evaluate the efficacy and safety of the use of Ningmitai capsule as an adjunctive stone expulsion therapy after RIRS. All patients were diagnosed with upper urinary tract calculi measuring 10–20 mm. The patients who successfully underwent RIRS were randomly assigned to the NMT capsule group (Ningmitai capsule, 1.52 g, three times daily) or the control group for 4 weeks based on the random number table method. The primary endpoints were the stone expulsion rate (SER) and stone-free rate (SFR). The average stone expulsion time (SET), average stone-free time (SFT) and complications were recorded. Between July 2, 2019, and December 17, 2020, 220 participants successfully underwent RIRS across 6 centers; 123 of them were randomized according to the exclusion criteria, and 102 (83%) were included in the primary analysis. The SERs on the 3rd, 7th, 14th and 28th days were significantly increased in the NMT capsule group compared with the control group (78.95% vs. 31.11%, 92.98% vs. 55.56%, 94.74% vs. 64.44%, 100% vs. 82.22%, respectively, *p* < 0.05). The SFRs on the 3rd and 7th days were not different (*p* > 0.05), while those on the 14th and 28th days were higher in the NMT capsule group (63.16% vs. 24.44% and 92.98% vs. 68.89%, *p* < 0.05). The average SET and average SFT of the NMT capsule group were remarkably shorter than those of the control group (*p* < 0.001). During the follow-up period, there were no significant differences in urine RBC counts between the two groups (*p* > 0.05). The urine WBC counts of the NMT capsule group were significantly lower than those of the control group on the 14^th^ day (*p* = 0.011), but there was no difference on the 3rd, 7th or 28th day (*p* > 0.05). The analgesic aggregate of the NMT capsule group was also much lower (*p* = 0.037). There were no significant differences in adverse events (*p* > 0.05), and they improved significantly without sequelae. This study indicated that NMT capsules can significantly promote stone clearance and are more effective and safer for upper urinary calculi after RIRS.

*Trial registration* Chinese Clinical Trial Registration No. ChiCTR1900024151.

*Date of registration* June 28, 2019.

**Supplementary Information:**

The online version contains supplementary material available at 10.1007/s00240-021-01296-w.

## Introduction

Upper urinary tract stones are one of the most common diseases in urology [1]. Major technological progress has been achieved for retrograde intrarenal surgery (RIRS), which is noninvasive, has a high-expulsion rate and has low morbidity, making it the preferred management strategy for upper urinary stones < 20 mm [2]. However, many factors, including patient characteristics, stone composition, location and size, technical level of the surgeon, and hardware facilities availability, must be considered when selecting the optimal treatment [2–5]. Even so, stone fragments and dust are still universal phenomena that would require further intervention if they fail to discharge spontaneously or lead to stone-related events. The wide range of stone-free rates (SFRs), from 45.6–100% [3, 6, 7], for stones in different locations of the upper urinary tract indicates that some patients still have small fragments or dust on follow-up studies, even those undergoing the most masterful RIRS procedure. Postprocedural residual fragments, which could cause infection, renal colic, stone recrudescence, and obstruction and eventually lead to renal failure [8–10], are the main risk factors for stone recurrence [11]. Therefore, increasing the SFR and accelerating the removal of residual stones has been a hot issue.

Ningmitai capsule (NMT) is a classic Miao nationality herba, per the National Drugs Standards (trial) WS-10348-(ZD-0348)-2002 [12], and is composed of Tou Hua Liao (*Herba Polygoni Capitati*), Bai Mao Gen (*Rhizoma Imperatae*), Da Feng Teng (*Radix Cocculi Trilobi*), Lian Qiao (*Fructus Forsythiae suspensae*), San Ke Zhen (*Berberidis radix*), Xian He Cao (*Herba Agrimoniae*) and Fu Rong Ye (*Folium Hibisci Mutabilis*) [13–17]. According to records in the traditional medicine literature, a calculus is caused by compact gravel. Coupled with dampness and heat, it develops into lithiasis, which is regarded as drenching syndrome by modern medicine [18].

Currently, the NMT capsule has been listed as a “National supervision recommended product” by the State Administration of Traditional Chinese Medicine (SATCHM). It not only has remarkable effects on heat clearance and detoxification, blood cooling and antileishmanial activity [19] but also shows broad-spectrum antibiosis and drug resistance reduction with fewer side effects after long-term administration. Consequently, NMT capsules as an adjunctive medicine alone or in combination with Western medicine have been widely applied in clinical practice to relieve symptoms related to expulsion [20, 21]. In addition, NMT capsules effectively prevent low back pain, hematuria, and urinary frequency symptoms after ureteroscopic holmium laser lithotripsy [22]. Encouraged by the excellent effect of NMT, we designed a multicenter, prospective, randomized controlled trial including 123 upper urinary stone patients across 6 centers to evaluate the effectiveness and safety of NMT capsules [23].

## Patients and methods

### Drug

Ningmitai capsule was supplied by Guiyang Xintian Pharmaceutical Co., Ltd. (16061806, Guiyang, Guizhou, China).

### Study design

This randomized controlled trial was designed by Urologists from the 6 centers of South China Base of Chinese Urolithiasis Federation in Henan Province, including the Second Affiliated Hospital of Zhengzhou University; Xinxiang Central Hospital; the 990th Hospital of the Joint Logistics Support Force of the Chinese People’s Liberation Army; the First Affiliated Hospital of Henan University of Science and Technology; Huaihe Hospital of Henan University; and Luoyang Central Hospital Affiliated with Zhengzhou University. This protocol was prospectively registered with the Chinese Clinical Trial Registry (ChiCTR1900024151). The investigators performed the data analysis at the Second Affiliated Hospital of Zhengzhou University. The Ethics Committee of the Second Affiliated Hospital approved the study protocol and informed consent form. Our study was performed across 6 centers between July 2019 and October 2020 (delayed by the COVID-19 epidemic). Per the study protocol, written informed consent was obtained from every patient. This trial was conducted in accordance with “The Declaration of Helsinki”, “The Guiding Principle for Drug Clinical Trial Data Management, Statistical Analysis, Planning and Reporting”, and “The Guidelines for Biostatistics in Drug Clinical Trials” and reported based on “The Consolidated Standards for Reporting Trials Statement”.

### Selection of subjects

All patients who were willing to be assessed for eligibility were recruited from the outpatient clinics. They were diagnosed with renal or upper ureteral calculi measuring 10–20 mm and received RIRS successfully.

### Inclusion criteria

- Patients aged 18–60 years with a diagnosis of upper urinary calculi, 10–20 mm in size, by IVP/CTU and noncontrast CT;
- Completion of RIRS by urologists with more than 100 cases of experience and proficiency in the RIRS operation;
- Successful ureteroscopic lithotripsy without perforation of the ureter, exfoliation of mucosa or injury to the kidney or bladder;
- Willingness to participate in this study and be followed up.

### Exclusion criteria

- Pregnancy, severe diabetes mellitus and renal insufficiency;
- History of previous ipsilateral surgery or natural lithotripsy;
- Patients with lower urinary tract obstruction;
- Patients with impacted or infectious stones;
- Prestenting patients;
- Use of alpha-1 receptor blockers or Chinese patented medicines that may affect the evaluation of the efficacy of lithotripsy within two weeks or allergy to experimental drugs;
- Failure to use drugs according to the prescription, which affects the judgment of its curative effect, or incomplete data, which affects the judgment of effectiveness and safety;
- Occurrence of serious adverse events in which it would not be appropriate to carry out the test again.

### Withdrawal criteria

Enrolled subjects meeting one of the following criteria were withdrawn from this study during the trial:

- Inability to meet the inclusion criteria or meeting the exclusion criteria during the follow-up period;
- Occurrence of other diseases that affected the efficacy and safety evaluation during the trial;
- Treatment not tolerated (Included in Safety Statistics);
- Self-discontinuation or coadministration of other therapeutic drugs, which would affect the efficacy review;
- The investigators did not consider it appropriate to continue the trial;
- The participants did not agree to continue to participate in this study (should be included in safety statistics if on medication);
- Loss to follow-up;
- Request for additional intervention, such as URS or ESWL, to remove stones before the end of the trial.

For patients who were withdrawn from the study, the case report form (CRF) was filled out with the reason for withdrawal, and the assessment and treatment records were revised as much as possible, with documentation of the date of the last follow-up. Close follow-up on withdrawals due to adverse events was conducted until the disappearance of adverse events.

### Randomization

Patients were assigned at a 1:1 ratio to receive the NMT or control treatment randomly. The statistical researcher produced randomization sequence (set seed = 20190606) with SPSS 25.0 to perform the randomization. He didn’t get involved in further study until the study analysis. Age, sex, stone side, stone size, stone composition and other stratification factors were collected for analysis. All randomly assigned patients were included in the efficacy and safety analysis.

### Procedures

All patients were successfully treated with RIRS. The stones were completely broken into fragments or dust less than 3 mm by the laser lithotriper with the mode of short pulse width, high-frequency (20–25 Hz) and low-energy (0.3–0.6 J) during the surgery. The prescription for the two groups of participants was as follows: after RIRS, all the patients in both groups were encouraged to drink 1–2 L water per day and to take part in some moderate activities. Besides, the patients in the NMT group were given four 0.38 g NMT capsules 3 times a day for up to 28 ± 7 days or they need other intervention. At the beginning of the register and next four follow-up visits, *V*_1_ (3 ± 1), *V*_2_ (7 ± 3), *V*_3_ (14 ± 3) and *V*_4_ (28 ± 7) days after RIRS, the following aspects of the two groups of participants were also assessed: (1) the general condition and urination details of the patient was observed; (2) low-dose non-contrast CT images were reviewed; (3) routine urine and culture tests were performed; (4) pain scores and analgesic medication rate were evaluated (the patients could use a 50-mg sodium diclofenac suppository if they cannot tolerate the pain); and (5) adverse events were recorded. Furthermore, we also provided patients with filters in advance to facilitate the collection of excreted stone fragments. They were asked to filter each urine discharge and collect the fragments. The urologists recorded the date when the residual fragments were first collected and send these fragments for composition analysis. For all patients removal of the stent was advised at 4 weeks. If stones were completely excluded over the course of treatment, the patient could stop medication use. In every center the baseline demographic and clinical data were collected before randomization. Patients could add a special Wechat account to feedback their response to the prescribed NMT.

### Outcomes

The investigators involved in the outcomes evaluation and statistical analysis of the study data were unaware of the subgroups.

The CT reports were issued by specialized imaging physicians, affiliated with the hospital’s imaging department, who had no knowledge of all the contents of this study. The primary endpoints of the analysis were the overall stone expulsion rate (SER) and stone-free rate (SFR). Stone expulsion was defined as the presence of stone fragments or dust in the urine after the operation. The first time of expulsion was defined as the stone expulsion time (SET). Stone-free status was confirmed by negative findings on low-dose non-contrast CT examination. The CT reports were issued by specialized imaging physicians, affiliated with the hospital's imaging department, who had no knowledge of all the contents of this study. The secondary endpoints included the time to stone expulsion and stone clearance, RBC count, WBC count, urine culture, analgesic medication rate, incidence of adverse events and other efficacy and safety parameters.

### Statistical analysis

Sampled data were compiled in a database specific for this study, and SPSS 25.0 software and GraphPad Prism 7 (GraphPad Software Inc., San Diego, CA, USA) were used to analyze the data. The measurement data are presented as means, standard deviations, medians, quartiles, maximum values and minimum values, while the counting data are presented as frequencies and percentages. The Kolmogorov–Smirnov test was applied to determine whether the data were normally distributed. If not, the Mann–Whitney *U* test was used for comparisons between groups; otherwise, the *t* test or Fisher’s exact test was used. *p* < 0.05 indicated a statistically significant difference.

## Results

**Fig. 1:**
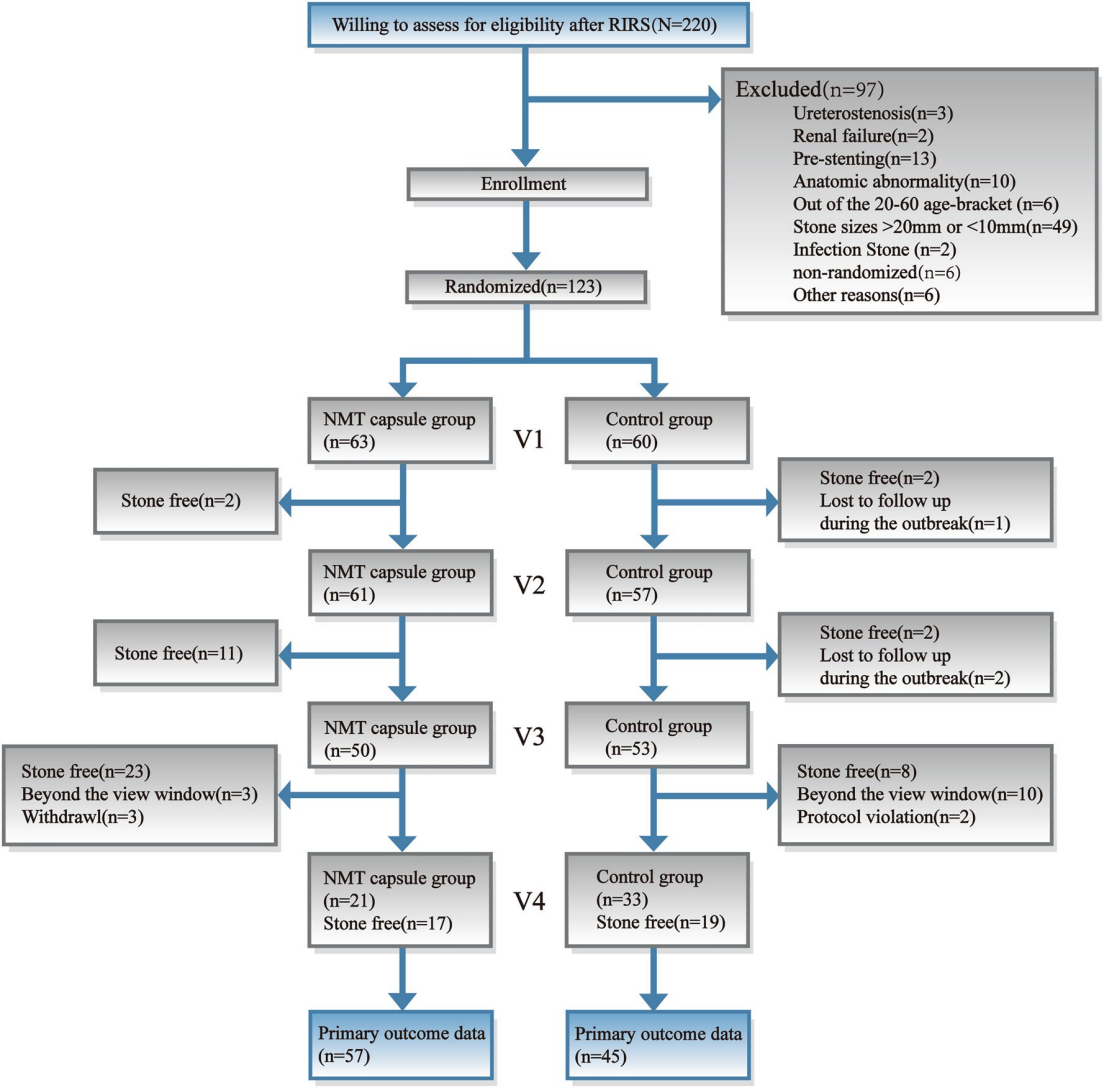
Trial profile. 220 patients, willing to assess for eligibility, were recruited from the outpatient departments of participating centers. They were diagnosed as renal or upper ureteral calculi from 10–20 mm and received Retrograde Intrarenal Surgery (RIRS) successfully. 97 of them were excluded according to the exclusion criteria. Among the remaining 123 were randomly assigned into the NMT capsule group (*n* = 63) or the control group (*n* = 60). In the end, there were 102 (83%) relative complete data being included in the primary outcome (57 in the NMT capsule,45 in the control). The control group patients were encouraged to drink 1–2 L water per day and to take part in some moderate activities; NMT group patients were not only encouraged to do like control group but also administrated to four capsules of NMT 0.38 g, taken 3 times daily up to a maximum of (28 ± 7) days or the need for intervention. At the beginning of the enrollment and the next four follow-up visits, *V*_1_ (3 ± 1), *V*_2_ (7 ± 3), *V*_3_ (14 ± 3) and *V*_4_ (28 ± 7) days after RIRS, two groups’ participants were requested to (1) observe the condition and urination; (2) review low dose non-contrast CT; (3) test urine routine and culture; (4) evaluate pain score; (5) record adverse events

A total of 220 participants underwent RIRS successfully. Ninety-seven of them were excluded because of ureterostenosis (*n* = 3), postoperative renal failure (*n* = 2), presenting (*n* = 13), anatomic abnormality (*n* = 10), being out of the age range (*n* = 6), stone sizes > 20 mm or < 10 mm (*n* = 49), infection due to stones (*n* = 2), nonrandomization (*n* = 6) and other reasons (*n* = 6). Among the remaining patients, 123 were randomly assigned to the NMT capsule group or the control group (63 in the NMT capsule group and 60 in the control group; Fig. 1). Then, after exclusion of patients who were lost to follow-up (*n* = 3), had agreement violations (*n* = 2), voluntarily withdrew (*n* = 3) and were beyond the follow-up window (*n* = 13) during treatment and follow-up, 102 patients were included in the analysis of the primary outcome (57 in the NMT capsule group and 45 in the control group; Fig. 1). The demographic and baseline characteristics, which included patient age, male sex, stone size, location, side, and main composition, revealed no statistically significant differences between the two groups (Table 1).

**Table 1:** Demographics and baseline characteristics

Variable	NMT capsule (*n* = 57)	Control (*n* = 45)	*p*
Male	40/57 (70.18)	29/45 (64.44)	0.539
Age (years)	45.51 ± 11.34	44.91 ± 9.99	0.781
Stone size (mm)	14.64 ± 2.96	14.48 ± 2.93	0.774
Stone location^b^			
Kidney	27/56 (48.21%)	23/45 (51.11%)	
Proximal ureter	24/56 (42.86%)	19/45 (42.22%)	0.955^a^
Both	5/56 (8.83%)	3/45 (6.67%)	
Stone side^b^			
Left	28/56 (50.00%)	25/45 (55.56%)	
Right	27/56 (48.21%)	19/45 (42.22%)	0.843^a^
Both	1/56 (1.79%)	1/45 (2.22%)	
Main stone composition			
Calcium oxalate	45/48 (93.75%)	35/41 (85.37%)	
Calcium phosphate	3/48 (6.25%)	4/41 (9.76%)	0.276^a^
Other	0	2/41 (4.88%)	

Data are presented as mean (standard deviation) or number (proportion)

^a^The method of statistics was Fisher’s exact test

^b^A stone side and location data was absent in NMT Capsule group

Next, we performed subgroup analyses of the NMT capsule for postoperative calculi exclusion to calculate the primary endpoints of the study, namely, the accumulative SER and accumulative SFR. The treatment outcomes are summarized in Table 2. The accumulative SERs for the 3rd, 7th, 14th and 28^th^ d were significantly increased in the NMT capsule group compared with the control group (78.95% vs. 31.11%, 92.98% vs. 55.56%, 94.74% vs. 64.44%, 100% vs. 82.22%, *p* < 0.05). The cumulative SFRs on the 14th and 28th days were significantly higher in the NMT capsule group than in the control group (63.16% vs. 24.44%, 92.98% vs. 68.89%, *p* < 0.05), while those on the 3rd and 7th days were not different between the two groups (3.17% vs. 3.33%, 20.63% vs. 6.67%, *p* > 0.05).

**Table 2:** Primary outcome results

Outcome	NMT capsule (*n* = 57)	Control (*n* = 45)	*p* value
Accumulative stone expulsion rate, *N* (%)			
* V*_1_ (3 ± 1 days)	45 (78.95)	14 (31.11)	< 0.001
* V*_2_ (7 ± 3 days)	53 (92.98)	25 (55.56)	< 0.001
* V*_3_ (14 ± 3 days)	54 (94.74)	29 (64.44)	< 0.001
* V*_4_ (28 ± 7 days)	57 (100.00)	37 (82.22)	0.001^a^
Stone expulsion patients			
Average stone expulsion time (days)	3.00 (1.00, 3.00)	6.00 (3.00, 17.50)	< 0.001^b^
Accumulative stone free rate, *N* (%)			
* V*_1_ (3 ± 1 days)	2 (3.51)	2 (4.44)	1.000^a^
* V*_2_ (7 ± 3 days)	13 (22.81)	4 (8.89)	0.061
* V*_3_ (14 ± 3 days)	36 (63.16)	11 (24.44)	< 0.001
* V*_4_ (28 ± 7 days)	53 (92.98)	31 (68.89)	0.002
Stone free patients			
Average stone free time (days)	14.00 (9.00, 23.00)	26.00 (14.00, 30.00)	0.006^b^
URBC counts			
* V*_1_ (3 ± 1 days)	122.00 (22.70, 1124.65), *n* = 57	422.35 (37.33, 2178.20), *n* = 45	0.152^b^
* V*_2_ (7 ± 3 days)	83.80 (12.25,557.05), *n* = 55	77.90 (19.35, 601.05), *n* = 43	0.927^b^
* V*_3_ (14 ± 3 days)	26.00 (0.50, 510.35), *n* = 44	58.00 (17.40, 682.15), *n* = 41	0.114^b^
* V*_4_ (28 ± 7 days)	16.80 (2.58, 753.70), *n* = 21	33.65 (11.60, 308.33), *n* = 34	0.407^b^
UWBC counts			
* V*_1_ (3 ± 1 days)	22.85 (8.65, 48.58), *n* = 57	29.00 (19.70, 68.80), *n* = 45	0.052^b^
* V*_2_ (7 ± 3 days)	17.45 (5.00, 36.28), *n* = 55	28.50 (7.80, 55.85), *n* = 43	0.285^b^
* V*_3_ (14 ± 3 days)	5.95 (1.48, 30.33), *n* = 44	18.80 (10.25, 38.50), *n* = 41	0.011^b^
* V*_4_ (28 ± 7 days)	15.90 (0.00, 43.00), *n* = 21	17.50 (6.15, 33.20), *n* = 34	0.562^b^
Analgesic medication rate, *N* (%)	7/54 (12.96)^c^	13/43 (30.23%)^c^	0.037

Nonnormal data are presented as median (interquartile range)

*URBC* urinary red blood cell, *UWBC* urinary white blood cell, *WMD* weighted mean difference

^a^The method of statistics was Fisher’s exact test

^b^The method of statistics was Mann–Whitney *U* test

^c^5 cases of analgesic treatment were missing

Considering the secondary end points, also shown in Table 2, the average stone expulsion time (SET) of the NMT capsule group was 3.00 (1.00, 3.00) days, while that of the control group was 4.00 (3.00, 11.00) days (*p* < 0.001). Among the stone-free patients, the average stone-free time (SFT) of the NMT capsule group was shorter than that of the control group (14.00 (9.00, 23.00) vs. 26.00 (14.00, 30.00), *p* = 0.006). During the follow-up period, the urine WBC counts of the NMT capsule group were significantly lower than those of the control group on day 14 (5.95 (1.48, 33.33) vs. 18.80 (10.25, 38.50), *p* = 0.011), but there were no differences among 3rd, 7th and 28th days (*p* > 0.05). Additionally, there were no significant differences in urine RBC counts between the two groups (*p* > 0.05). In terms of analgesic treatment, 5 cases were missing during the follow-up period, including 1 case in the control group and 4 cases in the NMT capsule group. Among the remaining 97 cases, the need for analgesic medication was significantly lower in the NMT capsule group than in the control group (12.95% (7/54) vs. 30.23% (13/43), *p* = 0.037).

Adverse events is shown in Table 3. Eleven adverse events were reported in 8 patients, including 5 cases of hematuria, 1 case of fever, 2 cases of pollakiuria, 2 cases of renal colic, 1 case of renal subcapsular hematoma and 1 case of low backache. All these patients had spontaneous recovery, and no significant difference in adverse events was found between the two groups (7.02% vs. 8.89%, *p* = 0.983).

**Table 3:** Adverse events

	NMT capsule (*n* = 57)		Control (*n* = 45)		*p*
	Time	Case (%)	Time	Case (%)	
Urinary tract symptoms	5	4 (7.02%)	6	4 (8.89%)	0.983
Systemic symptoms	0	0	0	0	

## Discussion

Urolithiasis is one of the most common urinary tract diseases. RIRS technology has become a popular option for the treatment of urolithiasis due to its safety advantage and less invasive properties [24, 25]. After RIRS, the stone fragments and dust normally tend to stay at the lower calyces under gravity. Patients were always advised to perform several traditional procedures to promote exclusion, such as drinking more water, standing upside down and increasing physical activity. However, these methods had little effect. These persistent residual fragments and dust could lead to morbidity and even require secondary surgical treatment for the patients in the future [26–29]. Therefore, accelerating stone discharge after RIRS has been a meaningful study in clinical practice.

Chinese doctors have unique experience in this respect. Specifically, there are calculus disease records in some well-known literature on traditional Chinese medicine from as early as more than two thousand years ago. The etiology of such diseases was considered a combination of dampness-heat and sandstone, blocking the waterway, failing to pass through and descending, and not dispersing blood stasis. Based on these theories, some herbs have been used as medical expulsion therapies since antiquity [30]. The NMT capsule is a classic Miao nationality herba for damp-heat accumulation, heat clearing and detoxification. Therefore, the purpose of this study was to explore the efficacy and safety of NMT capsules in promoting stone expulsion after RIRS.

The NMT capsule is a pure Chinese medicine preparation consisting of Touhualiao, Bai Mao Gen, Dafengteng, Lianqiao, Sankezhen, Xian He Cao and Furongye [31]. Regarding each ingredient, Tou Hua Liao, as the Monarch medicine, has a clearing-heat effect and promotes diuresis; Bai Mao Gen, Da Feng Teng and Lian Qiao, as the Minister, have cool-blood and detoxification effects; San Ke Zhen and Xian He Cao, as the Assistant, causes convergence and hemostasis; and Fu Rong Ye, as the Guide, enables the various drugs mentioned above to detoxify and promote detumescence.

In this study, the mean diameter of the stones, which was a basic factor affecting stone expulsion, was not significantly different between the two groups (14.48 ± 2.93 mm vs. 14.64 ± 2.96 mm, *p* = 0.774). All the stones were successfully fragmented into pieces by experienced senior doctors during the RIRS procedure. Another important factor was the composition of residual stones, comprising calcium oxalate and calcium phosphate. This was not significantly different either. For patients with residual stones after RIRS, continuous oral administration of NMT capsules could not only shorten the average time of expulsion and clearance but also improve the excretion rate (> 90% in the NMT capsule group). The mechanism we considered was the Tou Hua liaoin in the NMT capsule, which functions as a diuretic and relaxes smooth muscle [32].

Notably, our results also demonstrated that NMT capsules have obvious advantages in term of some of the postoperative complications. Often, during the process of fragment exclusion after RIRS, symptoms such as hematuria, infection, LUTS and pain can develop, especially in patients with a longtime indwelling stent [33, 34]. Some of these symptoms are similar to those of chronic prostatitis/chronic pelvic pain syndrome (CP/CPPS). NMT capsules have been widely used as a pharmacologic therapeutic method for CP/CPPS in the clinic. Some recently published RCTs have reported that NMT capsules can significantly improve pain symptoms and the quality of life of patients with CP/CPPS [35] and chronic epididymitis [36], and this is consistent with the findings from our study.

In our study, all patients were advised to remove the ureteric stent at 4 weeks. The proportion of patients who used analgesics for postoperative pain in the NMT capsule group was significantly lower than that in the control group. The urine WBC counts of the NMT capsule group were significantly lower than those of the control group on day 14. The urine RBC counts were not significantly different, although those of the NMT group were lower than those of the control group. We believe that the anti-inflammatory [37], analgesic [38] and antibacterial properties [13, 39, 40] of NMT capsules are due to the effect of each principal component. For instance, polysaccharides from Bai Mao Gen and hyperoside in Xian He Cao had anti-inflammatory effects via reduction of inflammation [41, 42]. Additionally, hyperoside can inhibit the transcription and expression of COX-2, resulting in pain relief [43]. Moreover, gallic acid from Tou Hua Liao, Bai Mao Gen, Lian Qiao, San Ke Zhen and Xian He Cao has significant antibacterial activity [44].

Given that the essential ingredients of NMT capsules, namely, Tou Hua Liao, Bai Mao Gen, Da Feng Teng and Lian Qiao, have diuretic and reducing ureteral resistance effects, we considered that the stone expulsion mechanism of NMT may increase the pressure above the stones via diuresis accompanied by a reduction in the resistance of the ureteral wall. However, further study is still needed to investigate its mechanism.

## Conclusions

Our data demonstrate that NMT capsules significantly promote stone expulsion after RIRS. Moreover, it is conducive to the prevention and treatment of postoperative pain, inflammation and infection.

## Supplementary Information

Below is the link to the electronic supplementary material.

Supplementary file1 (XLSX 36 kb)

## Data Availability

The datasets used or analysed during the current study are available from the corresponding author on reasonable request.

## Abbreviations

RIRS: Retrograde intrarenal surgery
NMT: Ningmitai capsule
SER: Stone expulsion rate
SFR: Stone free rate
SET: Stone expulsion time
SFT: Stone free time
RIRS: Retrograde intrarenal surgery
KUB: Kidney, ureter and bladder
IVP: Intravenous pyelography
CT: Computed tomography
CTU: Computed tomography urography
URS: Ureterorenoscopy
ESWL: Extracorporeal shock-wave lithotripsy
CRF: Case report form
WBC: White blood cell
RBC: Red blood cell
LUTS: Lower urinary tract symptoms
CP/CPPS: Chronic prostatitis/chronic pelvic pain syndrome

## Code availability

**S**ome or all data, models, or code generated or used during the study are available from the corresponding author by request.
